# Factors influencing COVID-19 mortality among cancer patients: A Brazilian multi-institutional study

**DOI:** 10.1371/journal.pone.0295597

**Published:** 2023-12-21

**Authors:** Jessé Lopes da Silva, Bruno Santos Wance de Souza, Lucas Zanetti de Albuquerque, Sabina Bandeira Aleixo, Gilmara Anne da Silva Resende, Daniela Galvão Barros de Oliveira, Emerson Neves dos Santos, Angélica Nogueira-Rodrigues, Renan Orsati Clara, Maria de Fatima Dias Gaui, Augusto Cesar de Andrade Mota, Vladmir Claudio Cordeiro de Lima, Daniela Dornelles Rosa, Rodrigo Ramella Munhoz, Igor Alexandre Protzner Morbeck, Ana Caroline Zimmer Gelatti, Clarissa Maria de Cerqueira Mathias, Andréia Cristina de Melo

**Affiliations:** 1 Division of Clinical Research and Technological Development, Brazilian National Cancer Institute, Rio de Janeiro, Rio de Janeiro, Brazil; 2 Department of Clinical Oncology, Galeao Air Force Hospital, Rio de Janeiro, Rio de Janeiro, Brazil; 3 Dasa Oncology, Hospital Nove de Julho, São Paulo, São Paulo, Brazil; 4 Department of Clinical Oncology, Evangelical Hospital of Cachoeiro de Itapemirim, Cachoeiro de Itapemirim, Espírito Santo, Brazil; 5 Department of Clinical Oncology, Amazonia Integrated Research Center, Manaus, Amazonas, Brazil; 6 Department of Clinical Oncology, Hospital Santa Izabel—Santa Casa da Bahia, Salvador, Bahia, Brazil; 7 Department of Clinical Oncology, Amil Integrated Oncology Care, São Paulo, Sao Paulo, Brazil; 8 Department of General Medicine UFMG, Federal University of Minas Gerais, Belo Horizonte, Minas Gerais, Brazil; 9 Brazilian Society of Clinical Oncology, São Paulo, São Paulo, Brazil; 10 Internal Medicine, Federal University of Rio de Janeiro, Rio de Janeiro, Rio de Janeiro, Brazil; 11 Department of Clinical Oncology, AMO Clinic–DASA, Salvador, Bahia, Brazil; 12 Department of Medical Oncology, A C Camargo Cancer Center, São Paulo, São Paulo, Brazil; 13 Department of Clinical Oncology, Hospital Moinhos de Vento, Porto Alegre, Rio Grande do Sul, Brazil; 14 Oncology Center—Hospital Sírio Libanes, São Paulo, São Paulo, Brazil; 15 Oncoclinicas Group of the Federal District, São Paulo, São Paulo, Brazil; 16 Oncoclinicas Group of Porto Alegre, Porto Alegre, Rio Grande do Sul, Brazil; 17 Brazilian Group of Thoracic Tumors, Porto Alegre, Rio Grande do Sul, Brazil; 18 Oncoclinicas Group of Bahia, Salvador, Bahia, Brazil; Children’s National Hospital, George Washington University, UNITED STATES

## Abstract

**Purpose:**

This study aimed to describe the demographic and clinical characteristics of cancer patients with COVID-19, exploring factors associated with adverse outcomes.

**Patients and methods:**

This retrospective cohort study methodically extracted and curated data from electronic medical records (EMRs) of numerous healthcare institutions on cancer patients diagnosed with a confirmed SARS-CoV-2 infection between May 2020 and August 2021, to identify risk factors linked to extended hospitalization and mortality. The retrieved information encompassed the patients’ demographic and clinical characteristics, including the incidence of prolonged hospitalization, acute complications, and COVID-19-related mortality.

**Results:**

A total of 1446 cancer patients with COVID-19 were identified (mean [Standard deviation] age, 59.2 [14.3] years). Most patients were female (913 [63.1%]), non-white (646 [44.7%]), with non-metastatic (818 [56.6%]) solid tumors (1318 [91.1%]), and undergoing chemotherapy (647 [44.7%]). The rate of extended hospitalization due to COVID-19 was 46% (n = 665), which was significantly impacted by age (p = 0.012), sex (p = 0.003), race and ethnicity (p = 0.049), the presence of two or more comorbidities (p = 0.006), hematologic malignancies (p = 0.013), metastatic disease (p = 0.002), and a performance status ≥ 2 (p = 0.001). The COVID-19-related mortality rate was 18.9% (n = 273), and metastatic disease (<0.001), performance status ≥2 (<0.001), extended hospitalization (p = 0.028), renal failure (p = 0.029), respiratory failure (p < 0.001), sepsis (p = 0.004), and shock (p = 0.040) significantly and negatively influenced survival.

**Conclusion:**

The rate of extended hospitalization and COVID-19-specific death in cancer patients was notably high and could be influenced by comorbidities, cancer treatment status, and clinical fragility. These observations may aid in developing risk counseling strategies regarding COVID-19 in individuals diagnosed with cancer.

## Introduction

Not long after epidemiological warnings were issued regarding clusters of patients with pneumonia of obscure origin, which was attributed to the extremely contagious and virulent severe acute respiratory syndrome coronavirus 2 (SARS-CoV-2), the emergence of COVID-19 was documented in December 2019 as an outbreak in Wuhan, situated in the Hubei province of China [1, 2]. By March 2020, this acute viral illness had been promptly identified as a pandemic due to its rapid dissemination across various regions worldwide [3]. Additionally, in March 2020, the initial occurrence of this disease in Brazil was detected in the state of São Paulo [4].

As per recent official estimates verified in January 2023, the worldwide tally of cases has already surpassed 664 million with over 6.71 million fatalities [5]. Concerning Brazil, the figures have surged to more than 36.5 million instances, and the distressing toll of fatalities associated with the disease has reached a staggering 695,000 [6]. Despite the considerable positive impact of mass vaccination and improved care protocols for critically ill patients, particularly in severe cases, there has been a recurrent surge in the epidemiological curve in Brazil due to the loosening of social isolation measures, low adherence of the populace to preventive measures, and the lack of a national governmental strategy for prevention [7].

Numerous large-scale reports have identified a range of comorbidities as important risk factors for COVID-19 mortality, including chronic obstructive pulmonary disorder (COPD), cardiovascular disease (CVD), diabetes, hypertension, obesity, neoplasia, acute kidney injury, and increased D -dimer [8]. Several studies have also established that patients with cancer are particularly prone to worse outcomes due to their pre-existing immunocompromised and hyperinflammatory state resulting from the underlying malignancy, advanced age, immunosuppressive anticancer treatments, frequent use of corticosteroids, lung impairment, and associated comorbidities [9–14]. It is worth noting that cancer patients represent a heterogeneous group with a diverse range of clinical conditions and treatment modalities, necessitating detailed profiling to establish the risk of severe outcomes or mortality from COVID-19 [15]. However, few national data describe the determinants that significantly impact the prognosis of cancer patients infected with SARS-CoV-2, thus highlighting the need for more comprehensive institutional protocols to be established for patients at higher risk of mortality.

The primary objective of the present investigation was to conduct an analysis of the demographic, clinical, and epidemiological characteristics of cancer patients afflicted with COVID-19 in Brazil. This was accomplished by pooling multi-institutional data through a task force established by the Brazilian Society of Clinical Oncology (SBOC), to elucidate the factors that have a consequential impact on the progression of COVID-19 toward more severe manifestations and ultimately lethal outcomes.

## Material and methods

### Study design and ethical considerations

This nationwide retrospective cohort study was conducted by retrieving curated data from electronic medical records (EMRs) across multiple institutions. The study encompassed adult patients aged 18 years and above who were diagnosed with cancer, regardless of whether it was a solid or hematological tumor, and who had a confirmed SARS-CoV-2 infection between May 2020 and August 2021. Patient information was inputted from June 30, 2020, to October 8, 2021, and was retrieved for analysis on March 30, 2023.

The diagnosis of COVID-19 was based on WHO guidance, which required confirmation through the positive result of a real-time reverse transcriptase polymerase chain reaction (RT-PCR) assay conducted on nasal and/or oropharyngeal swab specimens using the reagents and protocol established by the U.S. Centers for Disease Control and Prevention (CDC) [16, 17].

The study was authorized by the National Commission of Ethics in Research (CONEP) and adhered to the Good Clinical Practice guidelines. Given the retrospective nature of the research, written informed consent was not sought, and only de-identified data were evaluated.

### Data collection and outcomes

De-identified data were retrieved from the EMRs acquired by a task force operating under the aegis of the SBOC. The web-based data management system RedCap was utilized to construct the database, which received contributions from healthcare providers distributed throughout the country. Trained curators meticulously monitored the data’s quality on an ongoing basis.

The EMRs yielded a plethora of clinical and demographic information, including COVID-19’s initial course of illness, details on the patient’s cancer, and follow-up data. Specifically, age at the time of COVID-19 diagnosis, race (white vs. others), comorbidities, smoking and alcohol consumption, cancer site, staging, cancer status at the time of COVID-19 diagnosis, the time between cancer diagnosis and COVID-19 diagnosis, type of cancer treatment, adjustments in cancer treatment strategy due to COVID-19 diagnosis, delay or cessation of cancer treatment due to COVID-19, COVID-19 symptoms, laboratory tests upon diagnosis and during COVID-19’s course, the need of extended hospitalization, the development of acute complications, and death due to COVID-19 were all included.

### Statistical analysis

The baseline demographic characteristics of the participants included in the analysis were presented using descriptive statistics. Categorical data were expressed as absolute counts (N) and proportions (%), while continuous data were reported as means with standard deviation (SD) or medians with interquartile range (IQR), as determined by the Shapiro-Wilk test of normality. The co-primary endpoints were specified as follows: extended hospitalization, which refers to the need for hospitalization exceeding 24 hours, and death related to COVID-19 infection. The association between predictive factors and outcomes was assessed using logistic regression models. Results were reported as odds ratios (ORs) and their corresponding 95% confidence intervals (95% CI). Crude ORs were calculated for each predictive factor. Collinearity among the independent variables was verified using the Variance Inflation Factor (VIF). Multiple models were created using a forward step-by-step approach based on a conceptual hierarchical framework. Statistical significance was set at a p-value <0.05. Statistical analyses were conducted using R, version 4.2.1 (R Foundation for Statistical Computing). No imputation methods were employed to address missing variables.

## Results

The predetermined criteria for inclusion were met by 1446 cancer patients diagnosed with COVID-19 between May 2020 and August 2021 from 33 collaborating institutions spanning across 14 distinct states in Brazil. The demographic and clinical features of the patients are detailed in Table 1. The mean age of the patients was 59.2 years (standard deviation, SD 14.3). The cohort was predominantly composed of women (913 [63.1%]), non-white individuals (646 [44.7%]), and patients with a performance status of 0 or 1 (1178 [81.5%]). The study included 1318 (91.1%) patients with solid tumors and 128 (8.8%) with hematologic malignancies. The most common primary site of solid tumors was breast (504 [34.8%]), genitourinary (312 [21.6%]), and gastrointestinal (282 [19.5%]). Most patients had non-metastatic tumors (818 [56.6%]), with 647 (44.7%) patients undergoing chemotherapy, 150 (10.4%) receiving radiotherapy, 18 (1.2%) receiving immunotherapy, and 58 (4.0%) receiving molecular targeted therapy at the time of COVID-19 diagnostic confirmation. The majority of patients had 0 or 1 comorbidities (1178 [81.5%]), with hypertension being the most prevalent (555 [38.4%]). Current or former smokers accounted for 334 patients (23.1%), and current or former history of alcohol consumption was reported by 211 patients (14.6%).

**Table 1:** Univariate and multivariate analysis of baseline characteristics for mortality.

Total N = 1446 (%)	Outcome		*Univariate analysis*		*Multivariate analysis*	
	Alive n (%)	Death from COVID-19 n (%)				
			*OR (95%CI)*	*P* value	*OR (95%CI)*	*P* value
	1173 (81.1)	273 (18.9)				
*Variables*						
Mean age at COVID-19 diagnosis (SD)	58.3 (14.2)	63.4 (14.2)	1.03 (1.02–1.04)	<0.001	1.01 (0.99–1.03)	0.350
Sex						
Male	415 (35.4)	118 (43.2)	-			
Female	758 (64.6)	155 (56.8)	0.72 (0.55–0.94)	0.016		
Race and ethnicity						
Non-white	540 (56.4)	106 (65.8)	-			
White	417 (43.6)	55 (34.2)	0.67 (0.47–0.95)	0.026		
Asthma						
No	1158 (98.7)	272 (99.6)	-			
Yes	15 (1.3)	1 (0.4)	0.28 (0.02–1.41)	0.224		
Diabetes						
No	963 (82.1)	209 (76.6)	-			
Yes	210 (17.9)	64 (23.4)	1.40 (1.02–1.92)	0.036		
COPD						
No	1152 (98.2)	262 (96.0)	-			
Yes	21 (1.8)	11 (4.0)	2.30 (1.06–4.74)	0.027		
Chronic hepatopathy						
No	1167 (99.5)	271 (99.3)	-			
Yes	6 (0.5)	2 (0.7)	1.44 (0.21–6.27)	0.659		
Hypertension						
No	753 (64.2)	138 (50.5)	-			
Yes	420 (35.8)	135 (49.5)	1.75 (1.34–2.29)	<0.001		
Heart failure						
No	1151 (98.1)	259 (94.9)	-			
Yes	22 (1.9)	14 (5.1)	2.83 (1.40–5.55)	0.003		
Chronic kidney disease						
No	1153 (98.3)	259 (94.9)	-			
Yes	20 (1.7)	14 (5.1)	3.12 (1.52–6.21)	0.001		
Obesity						
No	1108 (94.5)	253 (92.7)	-			
Yes	65 (5.5)	20 (7.3)	1.35 (0.78–2.23)	0.260		
Number of comorbidities						
0 or 1	981 (83.6)	197 (72.2)	-			
≥ 2	192 (16.4)	76 (27.8)	1.97 (1.45–2.67)	0.001	1.24 (0.74–2.06)	0.412
Smoking						
Never	862 (76.4)	164 (71.0)	-			
Current / Former	267 (23.6)	67 (29.0)	1.32 (0.96–1.80).	0.086		
Alcohol consumption						
Never	930 (84.1)	171 (83.0)	-			
Current / Former	176 (15.9)	35 (17.0)	1.08 (0.72–1.59)	0.699		
Type of cancer						
Head and neck	26 (2.2)	7 (2.6)	-			
Gastrointestinal	227 (19.4)	55 (20.1)	0.90 (0.39–2.34)	0.815		
Lungs and thorax	70 (6.0)	21 (7.7)	1.11 (0.44–3.10)	0.826		
Sarcomas, skin, bone and connective tissue	56 (4.8)	16 (5.9)	1.06 (0.40–3.04)	0.908		
Breast	446 (38.0)	58 (21.2)	0.48 (0.21–1.25)	0.104		
Genitourinary	244 (20.8)	68 (24.9)	1.04 (0.45–2.68)	0.938		
Central nervous system	18 (1.5)	6 (2.2)	1.24 (0.35–4.34)	0.737		
Hematological	86 (7.3)	42 (15.4)	1.81 (0.76–4.84)	0.201		
Type of cancer						
Non-hematological	1087 (92.7)	231 (84.6)				
Hematological	86 (7.3)	42 (15.4)	2.30 (1.54–3.39)	<0.001		
Clinical stage						
Non-metastatic	275 (27.9)	108 (49.8)	2.55 (1.89–3.45)	<0.001	2.32 (1.49–3.64)	<0.001
Metastatic	709 (72.1)	109 (50.2)	-			
Current cancer management status at time of COVID-19 diagnosis						
Ongoing Surveillance in Remission	194 (16.7)	15 (5.5)	-			
Ongoing Surveillance with measurable disease	174 (14.9)	50 (18.4)	3.72 (2.06–7.07)	<0.001		
Neoadjuvant treatment	124 (10.6)	17 (6.2)	1.77 (0.85–3.72)	0.124		
Adjuvant treatment	284 (24.4)	29 (10.7)	1.32 (0.70–2.59)	0.401		
Palliative systemic treatment	261 (22.4)	110 (40.4)	5.45 (3.17–10.01)	<0.001		
Best supportive care	35 (3.0)	38 (14.0)	14.04 (7.13–28.97)	<0.001		
Performance status						
0 or 1	1013 (86.4)	145 (53.1)	-			
≥2	160 (13.6)	128 (46.9)	5.59 (4.18–7.48)	<0.001	2.62 (1.61–4.29)	<0.001
Time from cancer diagnosis to COVID-19 infection						
≤ 1 year	427 (64.0)	112 (71.3)	-			
> 5 years	240 (36)	45 (28.7)	0.71 (0.48–1.04)	0.084		
Chemotherapy						
No	658 (56.1)	141 (51.6)	-			
Yes	515 (43.9)	132 (48.4)	1.20 (0.92–1.56)	0.184		
Radiotherapy						
No	1059 (90.3)	237 (86.8)	-			
Yes	114 (9.7)	36 (13.2)	1.41 (0.93–2.09)	0.092		
Immunotherapy						
No	1158 (98.7)	270 (98.9)	-			
Yes	15 (1.3)	3 (1.1)	0.86 (0.20–2.62)	0.809		
Molecular target therapy						
No	1121 (95.6)	267 (97.8)	-			
Yes	52 (4.4)	6 (2.2)	0.48 (0.18–1.05)	0.097		

**Abbreviations:** COPD, chronic obstructive pulmonary disease; SD, standard deviation.

As shown in Table 1, the study observed a COVID-19-related mortality rate of 18.9% (273 patients). Univariate analysis demonstrated that deceased patients were significantly older than the living ones (63.4 vs. 58.3 years; p < 0.001). Females exhibited a 28% lower probability of mortality than males (p = 0.016). Additionally, patients with ≥ 2 comorbidities (p = 0.001), COPD (p = 0.027), essential hypertension (p < 0.001), and chronic kidney disease (p = 0.001) presented a higher risk of death. Patients with hematological malignancies (p < 0.001), PS ≥ 2 (p < 0.001), metastatic tumors (p < 0.001), ongoing surveillance with measurable disease (p < 0.001), palliative systemic treatment (p < 0.001), and those under best support care (p < 0.001) were also significantly associated with a higher mortality risk. However, by multivariate analysis, only the features of metastatic disease (p < 0.001) and performance status ≥2 (p < 0.001) significantly impacted survival.

Concerning the post-COVID-19 diagnosis characteristics (Table 2), the need to change cancer treatment protocols (p < 0.001), treatment delay (p = 0.008), or discontinuity of cancer treatment (p < 0.001) significantly and negatively impacted survival. Anosmia (p < 0.001), headache (p < 0.001), diarrhea (p = 0.044), dysgeusia (p < 0.001), dyspnea (p < 0.001), and myalgia (p < 0.001) at diagnosis were significantly associated with shorter survival. Moreover, COVID-19 complications such as bacterial coinfection (p < 0.001), anemia (p < 0.001), lymphopenia (p < 0.001), thrombocytopenia (p < 0.001), neutropenia (p = 0.007), high C-reactive protein (CRP) (p < 0.001), and increased lactate dehydrogenase (p < 0.001) negatively influenced survival. Nevertheless, according to the final hierarchical model, none of these variables had a significant impact on the mortality outcome.

**Table 2:** Univariate and multivariate analysis of post-COVID-19 diagnosis characteristics for mortality.

Total N = 1446 (%)	Outcome		*Univariate analysis*		*Multivariate analysis*	
	Alive n (%)	Death from COVID-19 n (%)				
			*OR (95%CI)*	*P* value	*OR (95%CI)*	*P* value
	1173 (81.1)	273 (18.9)				
Variables						
Cancer therapeutic strategy alteration due to COVID-19						
No	939 (80.9)	183 (69.3)	-			
Yes	221 (19.1)	81 (30.7)	1.88 (1.39–2.53)	<0.001		
Treatment delay						
No	976 (83.2)	245 (89.7)	-			
Yes	197 (16.8)	28 (10.3)	0.57 (0.37–0.85)	0.008		
Treatment interruption						
No	1156 (98.6)	225 (82.4)	-			
Yes	17 (1.4)	48 (17.6)	14.51 (8.36–26.38)	<0.001		
Anosmia						
No	898 (76.6)	263 (96.3)	-			
Yes	275 (23.4)	10 (3.7)	0.12 (0.06–0.22)	<0.001		
Headache						
No	898 (76.6)	249 (91.2)	-			
Yes	275 (23.4)	24 (8.8)	0.31 (0.20–0.48)	<0.001		
Diarrhea						
No	1053 (89.8)	256 (93.8)	-			
Yes	120 (10.2)	17 (6.2)	0.58 (0.33–0.96)	0.044		
Dysgeusia						
No	922 (78.6)	265 (97.1)	-			
Yes	251 (21.4)	8 (2.9)	0.11 (0.05–0.21)	<0.001		
Abdominal pain						
No	1123 (95.7)	259 (94.9)	-			
Yes	50 (4.3)	14 (5.1)	1.21 (0.64–2.17)	0.532		
Dyspnea						
No	802 (68.4)	93 (34.1)	-			
Yes	371 (31.6)	180 (65.9)	4.18 (3.17–5.55)	<0.001	1.37 (0.87–2.16)	0.178
Fever						
No	644 (54.9)	162 (59.3)				
Yes	529 (45.1)	111 (40.7)	0.83 (0.64–1.09)	0.184		
Myalgia						
No	902 (76.9)	243 (89.0)	-			
Yes	271 (23.1)	30 (11.0)	0.41 (0.27–0.61)	<0.001		
Bacterial coinfection						
No	1110 (94.6)	221 (81.0)	-			
Yes	63 (5.4)	52 (19.0)	4.15 (2.79–6.15)	<0.001		
Hemoglobin						
Low	245 (36.9)	144 (74.2)	4.93 (3.46–7.10)	<0.001		
Normal / High	419 (63.1)	50 (25.8)	-			
Platelets						
Low	100 (15.2)	60 (30.8)	2.48 (1.70–3.58)	<0.001		
Normal / High	557 (47.5)	135 (49.5)	-			
Lymphocytes						
Low	115 (20.0)	92 (52.9)	4.49 (3.13–6.45)	<0.001		
Normal / High	460 (80.0)	82 (47.1)	-			
Neutropenia						
No	565 (91.4)	151 (84.4)	-			
Yes	53 (8.6)	28 (15.6)	1.98 (1.20–3.21)	0.007		
C-reactive protein						
Normal	143 (49.8)	10 (8.5)	-			
High	144 (50.2)	108 (91.5)	10.72 (5.64–22.64)	<0.001		
Lactate dehydrogenase						
Normal	142 (65.4)	13 (19.4)	-			
High	75 (34.6)	54 (80.6)	7.86 (4.15–15.89)	<0.001		
D-dimer						
Normal	130 (58.8)	10 (15.6)	-			
High	91 (41.2)	54 (84.4)	7.71 (3.88–16.81)	<0.001		
Level of medical care						
Emergency room visit						
No	287 (24.5)	57 (20.9)	-			
Yes	886 (75.5)	216 (79.1)	0.39 (0.19–0.73)	0.006		
Telemedicine service						
No	1070 (91.2)	263 (96.3)				
Yes	103 (8.8)	10 (3.7)	0.39 (0.19–0.73)	0.006		
Extended hospitalization						
No	768 (65.5)	13 (4.8)	-			
Yes	405 (34.5)	260 (95.2)	37.93 (22.34–70.48)	<0.001	2.74 (1.14–6.98)	0.028
ICU						
No	305 (76.4)	111 (44.0)	-			
Yes	94 (23.6)	141 (56.0)	4.12 (2.94–5.81)	<0.001		
Severe complications and life support						
Heart failure						
No	1166 (99.4)	268 (98.2)	-			
Yes	7 (0.6)	5 (1.8)	3.11 (0.91–9.81)	0.054		
Liver failure						
No	1173 (100.0)	270 (98.9)	-			
Yes	0 (0.0)	3 (1.1)	NA	0.961		
Renal failure						
No	1159 (98.8)	221 (81.0)	-			
Yes	14 (1.2)	52 (19.0)	19.48 (10.91–37.09	<0.001	2.74 (1.14–7.04)	0.029
Respiratory failure						
No	1077 (91.8)	99 (36.3)	-			
Yes	96 (8.2)	174 (63.7)	19.72 (14.33–27.36	<0.001	6.86 (4.25–11.26)	<0.001
Sepsis						
No	1148 (97.9)	216 (79.1)	-		2.94 (1.41–6.28)	0.004
Yes	25 (2.1)	57 (20.9)	12.12 (7.49–20.13)	<0.001		
Shock						
No	1164 (99.2)	206 (75.5)				
Yes	9 (0.8)	67 (24.5)	42.06 (21.74–91.72)	<0.001	2.66 (1.08–7.08)	0.040
Vasoactive drugs						
No	1153 (98.3)	192 (70.3)				
Yes	20 (1.7)	81 (29.7)	24.32 (14.86–41.63	<0.001		
Renal replacement therapy						
No	1165 (99.3)	249 (91.2)	-			
Yes	8 (0.7)	24 (8.8)	14.04 (6.50–33.70	<0.001		
Mechanical ventilation						
No	1124 (95.8)	148 (54.2)	-			
Yes	49 (4.2)	125 (45.8)	19.37 (13.44–28.33)	<0.001		

**Abbreviations:** ICU, intensive care unity.

Regarding healthcare complexity level (Table 2), outpatients treated only by telemedicine (p = 0.006) or visiting the emergency room without requiring hospitalization (p = 0.006) had more prolonged survival. Conversely, patients in need of extended hospitalization (p < 0.001) or intensive care unit (p < 0.001) had significantly shorter survival. Furthermore, renal failure (p < 0.001), respiratory failure (p < 0.001), sepsis (p < 0.001), and shock (p < 0.001) were critical complications of COVID-19 that negatively influenced survival. The need for supportive vasoactive drugs (p < 0.001), renal replacement therapy (p < 0.001), and mechanical ventilation (p < 0.001) was also associated with shorter survival. By multivariate analysis, extended hospitalization (p = 0.028), renal failure (p = 0.029), respiratory failure (p < 0.001), sepsis (p = 0.004) and shock (p = 0.040) significantly and negatively influenced survival.

The occurrence rate of the extended hospitalization outcome was observed to be 46% (n = 665), as indicated in Table 3, which presents the data of the evaluated variables. Univariate analysis revealed that the mean age of hospitalized patients was significantly higher than that of outpatients (56.9 vs. 62.0 years; p = 0.001). Females were hospitalized at a lower rate than males (p < 0.001), while non-white patients were hospitalized less frequently than white patients (p < 0.001). Patients with two or more comorbidities (p < 0.001), including those with diabetes (p = 0.001), COPD (p = 0.012), essential hypertension (p < 0.001), heart failure (p = 0.001), chronic kidney disease (p < 0.001), and obesity (p = 0.015), exhibited a significant risk of prolonged hospitalization. Additionally, patients with breast cancer (p < 0.001), hematological malignancies (p < 0.001), metastatic tumors (p < 0.001), PS ≥2 (p < 0.001), evidence of measurable disease (p < 0.001), palliative systemic treatment (p < 0.001), treatment with molecular-targeted therapy (p = 0.002), or receiving best supportive care (p < 0.001) also demonstrated an increased risk of extended hospitalization. Ultimately, the final multiple hierarchical model validated that several factors had a significant effect on the probability of extended hospitalization, including age (p = 0.012), sex (p = 0.003), race and ethnicity (p = 0.049), the presence of two or more comorbidities (p = 0.006), hematologic malignancies (p = 0.013), metastatic disease (p = 0.002), and a performance status ≥ 2 (p = 0.001).

**Table 3:** Univariate and multivariate analysis of baseline characteristics for Extended hospitalization.

Total N = 1446 (%)	Outcome		*Univariate analysis*		*Multivariate analysis*	
	Alive n (%)	24-hour hospitalization n (%)				
			*OR (95%CI)*	*P* value	*OR (95%CI)*	*P* value
	781(54)	665(46)				
*Variables*						
Mean age at COVID-19 diagnosis (SD)	56.9 (13.8)	62.0 (14.5)	1.03 (1.02–1.03)	0.001	1.02 (1.00–1.03)	0.012
Sex						
Male	232 (29.7)	301 (45.3)	-			
Female	549 (70.3)	364 (54.7)	0.51 (0.41–0.63)	<0.001	0.58 (0.41–0.83)	0.003
Race and ethnicity						
Non-white	373 (53.2)	273 (65.5)	-			
White	328 (46.8)	144 (34.5)	0.60 (0.47–0.77)	<0.001	0.71 (0.51–1.00)	0.049
Asthma						
No	772 (98.8)	658 (98.9)	-			
Yes	9 (1.2)	7 (1.1)	0.91 (0.32–2.46)	0.857		
Diabetes						
No	669 (85.7)	503 (75.6)	-			
Yes	112 (14.3)	162 (24.4)	1.92 (1.47–2.52)	0.001		
COPD						
No	771 (98.7)	643 (96.7)	-			
Yes	10 (1.3)	22 (3.3)	2.64 (1.27–5.86)	0.012		
Chronic hepatopathy						
No	777 (99.5)	661 (99.4)	-			
Yes	4 (0.5)	4 (0.6)	1.18 (0.28–4.99)	0.820		
Hypertension						
No	536 (68.6)	355 (53.4)	-			
Yes	245 (31.4)	310 (46.6)	1.91 (1.54–2.37)	<0.001		
Heart failure						
No	772 (98.8)	638 (95.9)	-			
Yes	9 (1.2)	27 (4.1)	3.63 (1.76–8.23)	0.001		
Chronic kidney disease						
No	774 (99.1)	638 (95.9)	-			
Yes	7 (0.9)	27 (4.1)	4.68 (2.14–11.73)	<0.001		
Obesity						
No	746 (95.5)	615 (92.5)	-			
Yes	35 (4.5)	50 (7.5)	1.73 (1.11–2.72	0.015		
Number of comorbidities						
0 or 1	685 (87.7)	493 (74.1)	-			
≥ 2	96 (12.3)	172 (25.9)	2.49 (1.89–3.29,	<0.001	1.87 (1.20–2.92)	0.006
Smoking						
Never	590 (77.3)	436 (73.0)	-			
Current / Former	173 (22.7)	161 (27.0)	1.26 (0.98–1.61)	0.068		
Alcohol consumption						
Never	645 (84.6)	456 (82.9)	-			
Current / Former	117 (15.4)	94 (17.1)	1.14 (0.84–1.53)	0.398		
Type of cancer						
Head and neck	12 (1.5)	21 (3.2)	-			
Gastrointestinal	149 (19.1)	133 (20.0)	0.51 (0.24–1.06)	0.077		
Lungs and thorax	47 (6.0)	44 (6.6)	0.53 (0.23–1.20)	0.135		
Sarcomas, skin, bone and connective tissue	36 (4.6)	36 (5.4)	0.57 (0.24–1.32)	0.195		
Breast	354 (45.3)	150 (22.6)	0.24 (0.11–0.50)	<0.001		
Genitourinary	146 (18.7)	166 (25.0)	0.65 (0.30–1.35)	0.256		
Central nervous system	10 (1.3)	14 (2.1)	0.80 (0.27–2.37)	0.685		
Hematological	27 (3.5)	101 (15.2)	2.14 (0.92–4.85)	0.072		
Type of cancer						
Non-hematological	754 (96.5)	564 (84.8)	-			
Hematological	27 (3.5)	101 (15.2)	5.00 (3.27–7.89	<0.001	2.92 (1.26–6.92)	0.013
Clinical stage						
Non-metastatic	509 (75.5)	309 (58.6)	-			
Metastatic	165 (24.5)	218 (41.4)	2.18 (1.70–2.79)	<0.001	1.79 (1.23–2.59)	0.002
Current cancer management status at time of COVID-19 diagnosis						
Ongoing surveillance in remission	137 (17.7)	72 (10.9)	-			
Ongoing surveillance with measurable disease	95 (12.3)	129 (19.5)	2.58 (1.76–3.83)	<0.001		
Neoadjuvant treatment	89 (11.5)	52 (7.8)	1.11 (0.71–1.73	0.641		
Adjuvant treatment	213 (27.5)	100 (15.1)	0.89 (0.62–1.30	0.551		
Palliative systemic treatment	170 (22.0)	201 (30.3)	2.25 (1.59–3.21)	<0.001		
Best supportive care	6 (0.8)	67 (10.1)	21.25 (9.47–56.98)	<0.001		
Performance status						
0 or 1	725 (92.8)	433 (65.1)	-			
≥2	56 (7.2)	232 (34.9)	6.94 (5.10–9.58)	<0.001	4.02 (2.52–6.49)	0.001
Time from cancer diagnosis to COVID-19 infection						
≤ 1 year	273 (35.2)	266 (41.0)	-			
> 5 years	159 (20.5)	126 (19.4)	0.81 (0.61–1.08)	0.160		
Chemotherapy						
No	431 (55.2)	368 (55.3)	-			
Yes	350 (44.8)	297 (44.7)	0.99 (0.81–1.22)	0.954		
Radiotherapy						
No	709 (90.8)	587 (88.3)	-			
Yes	72 (9.2)	78 (11.7)	1.31 (0.93–1.84)	0.119		
Immunotherapy						
No	769 (98.5)	659 (99.1)	-			
Yes	12 (1.5)	6 (0.9)	0.58 (0.20–1.51)	0.284		
Molecular target therapy						
No	738 (94.5)	650 (97.7)	-			
Yes	43 (5.5)	15 (2.3)	0.40 (0.21–0.70)	0.002		

**Abbreviations:** COPD, chronic obstructive pulmonary disease.

Further details on the construction of hierarchical models are provided in the supplementary materials (S1 Table).

## Discussion

This large multicenter cohort provides an accurate depiction of the characteristics of cancer patients diagnosed with COVID-19 in Brazil during the peak of the pandemic. The study focuses on the impact of this viral infection on extended hospitalization and patient survival. The findings of this study provide detailed insights into several significant aspects of the COVID-19 disease course in patients with pre-existing cancer. Notably, females were overrepresented, with breast cancer comprising more than one-third of cases, and chemotherapy was being administered in almost half of the patients. Hypertension was the most frequently observed comorbidity, with nearly one-quarter of patients reporting a history of smoking or alcohol consumption. Nearly half of the patients required an extended hospital stay, and almost one-fifth of the patients had a fatal outcome.

COVID-19 pandemic has raised significant challenges in the management of cancer patients. Many recommendations focused on the treatment of both cancer and COVID-19 in infected cancer patients, as well as reducing the risk of virus contraction in uninfected cancer patients [18–20]. However, evidence regarding the initiation of chemotherapy in COVID-19 patients is mixed. While some studies have found no association between chemotherapy administration and severe or critical COVID-19 illness in cancer patients, other studies have reported higher odds of in-hospital COVID-related death in patients with hematologic malignancies who had recently undergone chemotherapy [12, 21, 22]. Therefore, making definitive suggestions for chemotherapy treatment in cancer patients with COVID-19 infection is challenging. Another area of concern is the delay in cancer treatment during the pandemic. While some patients have requested delays in their treatments, guidelines generally recommend standard treatments to avoid worsening cancer-related prognosis, with exceptions made for postponing treatment for lung cancer patients infected with COVID-19 [20]. However, delays in certain treatments may have unknown effects, particularly for cancer patients using multiple therapies at once. Therefore, careful consideration is necessary when making treatment decisions for cancer patients during the COVID-19 era.

The collective COVID-19-related mortality rate observed in this study was comparably inferior to the corresponding rates reported in other series that encompassed cancer patients [10–12, 23]. However, Chavez-MacGregor et al [15], utilizing population-based data, presented a COVID-19-associated death toll of 7.8% for individuals with recent cancer treatment. Nevertheless, all of these documented mortality rates were significantly greater than those reported for non-cancer patients [24]. It is noteworthy that a subset of patients received a classification of non-invasive support after, or even preceding, the diagnosis of COVID-19, due to the severity of their advanced malignancies. This may have potentially led to an overestimation of the proportion of deaths caused by COVID-19. Additionally, a considerable proportion of the patients under evaluation were undergoing non-curative treatment or optimal supportive care during the study period, indicating the advanced stage of their respective underlying diseases.

Similar to the multicenter studies reported by Mehta et al [11] and Dai et al [25], individuals with hematologic malignancies exhibited a greater death rate attributed to COVID-19 compared to those with solid tumors. This observation is likely due to the administration of more myelosuppressive therapy in these patients, who are frequently immunocompromised by their underlying condition. Growing evidence suggests that a significant pathogenic mechanism may involve cytokine storm syndrome resulting from hyperinflammation, leading to pulmonary damage. Patients with hematologic malignancies may be more susceptible to cytokine-mediated inflammation as a consequence of perturbations in the myeloid and lymphocyte cell compartments [26, 27]. Jee et al [21] conducted a retrospective cohort study involving 309 cancer patients, which revealed that lung and hematologic cancer patients had hazard ratios of 2.0 and 1.90, respectively, for developing severe or critical COVID-19 events.

The presence of metastases and a performance status score of ≥ 2 were identified as predictors of increased mortality risk in this cohort. Prior investigations have postulated a correlation between the severity of COVID-19 and the presence of cancer metastases [28–30]. Patients with metastatic disease generally exhibit a more compromised systemic status, rendering them more susceptible to severe viral infections. Furthermore, Mohiuddin and Kasahara [31] propose that dysregulation in the pro-inflammatory mechanisms and signal transduction pathways, particularly involving vascular endothelial growth factor (VEGF), transforming growth factor (TGF)-β signaling pathways, matrix metalloproteinases (MMPs), and epiregulin (EREG), may contribute to this association.

Some serum biomarkers, including anemia, lymphopenia, thrombocytopenia, elevated levels of CRP, lactate dehydrogenase, and D-dimer, may be associated with mortality [32, 33]. It is therefore advisable to develop treatment guidelines based on this emerging evidence, as certain biomarkers may indicate severe COVID-19 illness in cancer patients. CRP, a marker of endothelial dysfunction during inflammation due to infection and chronic cardiovascular disorders, is an essential indicator of the severity of COVID-19 infection, as demonstrated by multiple studies [34–36]. In COVID-positive cancer patients, CRP levels may be especially useful in prognosis, as they have been shown to be significantly correlated with higher odds of COVID-related mortality [37]. Thus, CRP should be considered a prognostic marker during the treatment of COVID-19 illness in cancer patients.

Liu and Hill [38] have suggested an association between COVID-19 severity and "cytokine storm syndromes" resulting from innate immune system activation. Primary immunodeficiency (PID) patients, with compromised immune systems, have an increased susceptibility to severe COVID-19 infections. Additionally, PID patients deficient in antiviral or interferon immune signaling may further heighten their risk of severe COVID-19 manifestations. As suggested by Liu et al. [39], measuring pro-inflammatory cytokine levels in serum holds potential for various clinical management applications in COVID-19, including risk assessment, disease monitoring, prognosis determination, therapy selection, and treatment response prediction. However, the interpretation of multiplex cytokine data in COVID-19 patients is challenging due to the involvement of cytokines in various immunological disorders and diseases. Moreover, the commutability of cytokine immunoassays can be affected by biological and technical variables, thereby complicating cytokine data interpretation.

Interleukin-6 (IL-6), a proinflammatory cytokine that plays a key role in fever and acute immune response, dictates the release of CRP from the liver [35]. It has a reliable prognostic value during severe COVID-19 infection and is correlated with cancer [40–42]. Blocking IL-6 has been shown to be beneficial in cancer patients when combined with other conventional therapies [43]. Tocilizumab, an antibody that blocks IL-6, reduces inflammation and decreases the mortality rate in COVID-19 patients. However, blocking this pathway may lead to an immunocompromised state, increasing the chances of secondary infection or causing off-target effects [44]. The potential risks of tocilizumab use are illustrated by a case report of two COVID-19 patients who received tocilizumab and progressed to hemophagocytic lymphohistiocytosis, a highly fatal disease characterized by overproduction of immune cells [45]. Due to IL-6’s independent correlation with cancer and COVID-19 infection, as well as its close relation to CRP, IL-6 should be considered a potential prognostic indicator for cancer patients with COVID-19 illness, but further trials are needed [46].

Certain subgroups of cancer patients are prone to increased risks of unfavorable outcomes. Early data from non-cancer patients indicated that only a small proportion of cases progressed with severe complications, including septic shock, respiratory failure, acute kidney and multiple organ failure [25, 47]. However, the present study highlights significantly higher rates of such complications in cancer patients, emphasizing their increased susceptibility. Compared to Kuderer et al ’s international prospective series that examined over 928 patients, the odds of COVID-related complications were heterogeneous [13]. Specifically, the ICU admission rate was 14% (in contrast to 36.1% in the current study) and the mechanical ventilation requirement rate was 12% (similar to the current study). Consistent with previous research, this retrospective analysis strongly implies that complications such as renal and respiratory failure, sepsis, and shock may be linked to shorter survival [15, 48–50].

The current study found a 46% rate of severe illness requiring extended hospitalization, which is similar to rates reported in previous studies by Chavez-MacGregor et al (33.7%) [15] and Kuderer et al (50.0%) [13]. In the TERAVOLT registry, which included patients with thoracic malignant neoplasms and COVID-19 from multiple countries, the hospitalization rate was much higher at 76%, with a COVID-19-related mortality rate of 33% [51]. The risk of extended hospitalization was found to be increased in patients with two or more comorbidities, hematological malignancies, metastatic disease, and those with a performance status of 2 or higher, which is consistent with previous reports [13, 15, 51]. Therefore, it is important to consider these factors when assessing the risk of severe illness and hospitalization in cancer patients with COVID-19.

The present investigation exhibits notable strengths, including the use of a large and diverse dataset collected from multiple institutions, which represents one of the largest Brazilian series exploring the impact of SARS-CoV-2 infection on cancer patients. The analysis encompassed a wide range of variables, allowing for the exploration of potential associations with the risk of extended hospitalization and mortality.

However, some significant limitations that must be acknowledged. Firstly, the study relied on EHRs to identify COVID-19 cases, which may have introduced ascertainment bias if diagnostic tests were not reported within the EMR system. Additionally, cancer patients, especially those who had recently undergone treatment, were more likely to undergo COVID-19 testing, regardless of symptoms, which may have introduced selection bias. Patients who were not on chemotherapy may have stopped treatment due to poor performance status, which could increase their risk of death from COVID-19 and impair our ability to assess the actual risk of anti-cancer treatments in a population with better performance status. However, this limitation was attempted to be addressed through multivariate analyses with age and comorbidity corrections.

Another limitation of this retrospective study was the high rate of missing data for some variables. Furthermore, the absence of a comparison group of non-cancer patients with COVID-19 or cancer patients without COVID-19 hinders the ability to compare morbidity and mortality outcomes. Finally, the results are reflective of adult patients treated in Brazil and may not generalize to other settings of countries with different resource profiles.

## Conclusion

In summary, this multicenter cohort study conducted in Brazil during the peak of the COVID-19 pandemic has highlighted the significant challenges posed by the pandemic on cancer patients. The study’s findings indicate that COVID-19 infection in patients with pre-existing cancer is associated with extended hospital stays, higher fatality rates, delays in cancer treatment, and challenges in administering chemotherapy. Notably, metastases and poor performance status were identified as risk factors for increased mortality risk. These findings underscore the importance of careful consideration of treatment decisions for cancer patients during the COVID-19 era to avoid worsening cancer-related prognoses. This study’s contributions provide critical insights into the management of cancer patients during the COVID-19 pandemic, highlighting the need for vigilant care to mitigate the impact of COVID-19 on cancer patients’ outcomes.

## Supporting information

S1 Dataset (XLTX)

S1 Table Multiple logistic regression for mortality (hierarchical approach) (n = 1195). Note: Model 1: Baseline variables; Model 2: Cancer variables; Model 3: Signs and symptoms at COVID-19 diagnosis; Model 4: Hospitalization related outcomes.
(DOCX)
