# Supplementary material for: Factors influencing COVID-19 mortality among cancer patients: A Brazilian multi-institutional study
Source: PLoS One. 2023 Dec 21;18(12):e0295597. doi: 10.1371/journal.pone.0295597 (PMC10734930; doi:10.1371/journal.pone.0295597)
Supplement: S1 Table — Note: Model 1: Baseline variables; Model 2: Cancer variables; Model 3: Signs and symptoms at COVID-19 diagnosis; Model 4: Hospitalization related outcomes. (DOCX) [file pone.0295597.s002.docx]

S1 Table. Multiple Logistic Regression for mortality (hierarchical approach) **n=1195**

| Total n = 1446 (%) | *Model 1*  *OR (95%CI, p)* | *Model 2*  *OR (95%CI, p)* | *Model 3*  *OR (95%CI, p)* | *Model 4*  *OR (95%CI, p)* |
| --- | --- | --- | --- | --- |
| *Variables* |  |  |  |  |
| Age ate COVID-19 diagnosis | 1.02 (1.01-1.03, <0,001) | 1.02 (1.00-1.03, p=0.017) | 1.02 (1.00-1.03, p=0.027) | 1.01 (0.99-1.03, p=0.350) |
| Number of comorbidities |  |  |  |  |
| 0 or 1 | - | - | - | - |
| ≥ 2 | 1.60 (1.15-2.20, 0.004) | 1.60 (1.08-2.36, p=0.017) | 1.38 (0.89-2.12, p=0.147) | 1.24 (0.74-2.06, p=0.412) |
| Clinical stage |  |  |  |  |
| Non-metastatic | - | - | - | - |
| Metastatic |  | 1.88 (1.35-2.60, p<0.001) | 1.93 (1.34-2.78, p<0.001) | 2.32 (1.49-3.64, p<0.001) |
| Performance status |  |  |  |  |
| 0 or 1 |  | - | - | - |
| ≥2 |  | 3.81 (2.65-5.48, p<0.001) | 2.90 (1.92-4.39, p<0.001) | 2.62 (1.61-4.29, p<0.001) |
| Dyspnea |  |  |  |  |
| No |  |  | - | - |
| Yes |  |  | 3.67 (2.56-5.31, p<0.001) | 1.37 (0.87-2.16, p=0.178) |
| Bacterial coinfection |  |  |  |  |
| No |  |  | - | - |
| Yes |  |  | 0.47 (0.24-0.88, p=0.020) |  |
| Level of Medical Care |  |  |  |  |
| Hospitalization for more than 24 hours |  |  |  |  |
| No |  |  |  | - |
| Yes |  |  |  | 2.74 (1.14-6.98, p=0.028) |
| Renal failure |  |  |  |  |
| No |  |  |  | - |
| Yes |  |  |  | 2.74 (1.14-7.04, p=0.029) |
| Respiratory failure |  |  |  |  |
| No |  |  |  | - |
| Yes |  |  |  | 6.86 (4.25-11.26, p<0.001) |
| Sepsis |  |  |  |  |
| No |  |  |  | - |
| Yes |  |  |  | 2.94 (1.41-6.28, p=0.004) |
| Shock |  |  |  |  |
| No |  |  |  | - |
| Yes |  |  |  | 2.66 (1.08-7.08, p=0.040) |

**Note:** Model 1: Baseline variables; Model 2: Cancer variables; Model 3: Signs and symptoms at COVID-19 diagnosis; Model 4: Hospitalization related outcomes
